# Supplementary material for: The surprising role of the default mode network in naturalistic perception
Source: Commun Biol. 2021 Jan 19;4:79. doi: 10.1038/s42003-020-01602-z (PMC7815915; doi:10.1038/s42003-020-01602-z)
Supplement: Supplementary file 3 — Description of Supplementary Files [file 42003_2020_1602_MOESM3_ESM.pdf]

Description of Additional Supplementary Files

File name: Supplementary Data

Description: Source data underlying Figures 2,3,4. Sheet 1 titled “Figure 2”: SFPA correlation values by region pair; Sheet 2 titled “Figure 3”: ISFC values around cognitive peaks, for each network, behavioral measure, and time-bin; Sheet 3 titled “Figure 4”: SFPA correlation values by region pair.
