# Supplementary material for: The surprising role of the default mode network in naturalistic perception
Source: Commun Biol. 2021 Jan 19;4:79. doi: 10.1038/s42003-020-01602-z (PMC7815915; doi:10.1038/s42003-020-01602-z)
Supplement: Supplementary file 5 — Reporting Summary [file 42003_2020_1602_MOESM5_ESM.pdf]

Corresponding author(s): Talia Brandman
Last updated by author(s): Nov 8, 2020

Reporting Summary

Nature Research wishes to improve the reproducibility of the work that we publish. This form provides structure for consistency and transparency in reporting. For further information on Nature Research policies, see our Editorial Policies and the Editorial Policy Checklist.

Statistics

For all statistical analyses, confirm that the following items are present in the figure legend, table legend, main text, or Methods section.

n/a	Confirmed	
☐	☒	The exact sample size (n) for each experimental group/condition, given as a discrete number and unit of measurement
☐	☒	A statement on whether measurements were taken from distinct samples or whether the same sample was measured repeatedly
☐	☒	The statistical test(s) used AND whether they are one- or two-sided<br>*Only common tests should be described solely by name; describe more complex techniques in the Methods section.*
☐	☒	A description of all covariates tested
☐	☒	A description of any assumptions or corrections, such as tests of normality and adjustment for multiple comparisons
☐	☒	A full description of the statistical parameters including central tendency (e.g. means) or other basic estimates (e.g. regression coefficient) AND variation (e.g. standard deviation) or associated estimates of uncertainty (e.g. confidence intervals)
☐	☒	For null hypothesis testing, the test statistic (e.g. F, t, r) with confidence intervals, effect sizes, degrees of freedom and P value noted<br>*Give P values as exact values whenever suitable.*
☒	☐	For Bayesian analysis, information on the choice of priors and Markov chain Monte Carlo settings
☐	☒	For hierarchical and complex designs, identification of the appropriate level for tests and full reporting of outcomes
☐	☒	Estimates of effect sizes (e.g. Cohen's d, Pearson's r), indicating how they were calculated

Our web collection on statistics for biologists contains articles on many of the points above.

Software and code

Policy information about availability of computer code

Data collection	We collected behavioral responses via Amazon Mechanical Turk, in a html-CSS-JavaScript custom questionnaire. Open source data was obtained with no software.
Data analysis	Data were analyzed using MATLAB (MathWorks) with statistical parametric mapping (SPM) for preprocessing, NeuroElf for region-of-interest (ROI) organization and BrainNet for ROI visualization.

For manuscripts utilizing custom algorithms or software that are central to the research but not yet described in published literature, software must be made available to editors and reviewers. We strongly encourage code deposition in a community repository (e.g. GitHub). See the Nature Research guidelines for submitting code & software for further information.

Data

Policy information about availability of data

All manuscripts must include a data availability statement. This statement should provide the following information, where applicable:
- Accession codes, unique identifiers, or web links for publicly available datasets
- A list of figures that have associated raw data
- A description of any restrictions on data availability

All materials, code and data directly collected for this study will be made freely available upon request.

Field-specific reporting

Please select the one below that is the best fit for your research. If you are not sure, read the appropriate sections before making your selection.

☐ Life sciences ☒ Behavioural & social sciences ☐ Ecological, evolutionary & environmental sciences

For a reference copy of the document with all sections, see nature.com/documents/nr-reporting-summary-flat.pdf

Behavioural & social sciences study design

All studies must disclose on these points even when the disclosure is negative.

Study description	Quantitative experimental study of naturalistic perception, matching patterns of cognitive-state fluctuations with fMRI inter-regional and inter-subject coactivations during movie viewing. Cognitive states were measured behaviorally via online questionnaires, and fMRI responses to the same movies were obtained with permission from the groups that originally recorded them in previous studies.
Research sample	Data collected directly for this study included participants who are registered as "workers" of Amazon Mechanical Turk, within the US. Forty-five participants (19 female, age 33.2 ± 8.7 years) were included in the behavioral data for the movie Sherlock, and 41 participants (17 female, age 31.3 ± 7.7 years) were included in the behavioral data for the movie Bang! You're Dead. All participants reported normal or corrected-to-normal vision and hearing and fluent English. fMRI data for Sherlock included 17 participants obtained with permission from Chen et al. (2016) and 18 participants obtained with permission from Zadbood et al. (2017). fMRI data collection and sharing for Bang! You're Dead was provided by the Cambridge Centre for Ageing and Neuroscience (CamCAN; Shafto et al., 2014; Taylor et al., 2017).
Sampling strategy	Participants on Amazon Mechanical Turk performed the task at will, and were allowed to join until the predefined quota was filled. For Sherlock, we utilized the entire fMRI datasets received. For Bang! You're dead, from the CamCAN repository data, we randomly sampled 30 participants within an age range of 20-50 years, to fit within the same range as our behavioral participants. Sample sizes were chosen based on the authors' previous experience with these experimental modalities (e.g. behavioral Amazon Mechanical Turk data in Brandman & Peelen, 2017; fMRI inter-subject coactivations during movie viewing in Simony et al., 2016). All sample sizes were predetermined and no additional data were collected post hoc.
Data collection	Participants were first screened for technical compatibility (e.g. operating system, internet connection, screen size and sound) and fluent English writing ability, in order to enable successful video viewing and questionnaire completion. In addition, participants ability to properly hear and see the video was tested before beginning the experiment, in a short audiovisual clip followed by auditory and visual catch questions. Participants were instructed to sit at a distance of 1 foot (12 inches) from the screen. Sherlock was presented at 200 mm over 112.5 mm, and Bang! You're Dead was presented at 180 mm over 135 mm. Participants viewed the movie from start to end without pausing, skipping or rewinding. Single continuous viewing was additionally monitored via recorded viewing times. After viewing the movie, participants first typed a brief free recall describing the content of the movie. Next, participants completed a questionnaire recording their self-reported experience referring to various events of the movie. The questionnaire for Sherlock referred to 49 events, sampling the time-course of the movie at intervals of ~30 sec. The questionnaire for Bang! You're Dead referred to 39 events, sampling the movie at intervals of ~15 seconds. Each participant was probed on one third of the total events in the movie. Included events were probed in random order throughout the questionnaire. The reminder for each event was presented as a timestamp with a short description of something that happened at a particular moment in the movie (e.g. 10:14 - Sherlock (in lab): "Mike, can I borrow your phone?"). Participants were then asked to focus their memory on that particular event, including no more than a few seconds before and after it. They rated how vividly they remembered the event, typed a detailed free recall of the event, and rated to what extent the event was surprising, emotionally intense, emotionally negative or positive, and important to the plot. All ratings were collected on scales from 1 to 7. Instructions for the free recall of each event resembled the autobiographical interview method, asking participants to recall every detail they remembered about what happened at that moment of the movie, what they saw and heard, their thoughts, emotions and physical sensations while viewing the event.
Timing	Behavioral data were collected in two short periods, one for each movie. The first, throughout October 2018, and the second throughout January 2019. The time in between was used for data analysis of the first movie, of both behavioral and fMRI data, and particularly for the initial development of our new technique of state-fluctuation pattern matching (SFPA) that we report in the manuscript.
Data exclusions	Two additional participants for Sherlock, and 3 additional participants for Bang! You're Dead, were excluded from behavioral-data analysis because they did not complete the task as instructed.
Non-participation	On Amazon Mechanical Turk participants opt in at will, and their responses are received only if they complete the task in full. We thus have no information about participants who had decided not to opt in or who had quit the task without completing it.
Randomization	The behavioral questionnaires were divided into 3 subsets of questions (chronologically interleaved) due their length, to which participants were randomly assigned.

Reporting for specific materials, systems and methods

We require information from authors about some types of materials, experimental systems and methods used in many studies. Here, indicate whether each material, system or method listed is relevant to your study. If you are not sure if a list item applies to your research, read the appropriate section before selecting a response.

Materials & experimental systems

n/a	Involved in the study
x	Antibodies
x	Eukaryotic cell lines
x	Palaeontology and archaeology
x	Animals and other organisms
	x Human research participants
x	Clinical data
x	Dual use research of concern

Methods

n/a	Involved in the study
x	ChIP-seq
x	Flow cytometry
	x MRI-based neuroimaging

Human research participants

Policy information about studies involving human research participants

Population characteristics	See above.
Recruitment	Amazon Mechanical Turk participants chose to join the task at will, and received payment for participation. The potential sample was therefore large and heterogeneous. Taken together with the naturalistic nature of the task (watch a movie and answer questions), no particular biases are expected to have affected the sample.
Ethics oversight	Experimental procedures for data collected in this study were approved by the institutional review board (IRB; approval reference # 533-2) of the Weizmann Institute of Science. All participants gave informed consent.

Note that full information on the approval of the study protocol must also be provided in the manuscript.

Magnetic resonance imaging

Experimental design

Design type	Movie viewing, and resting state.
Design specifications	Sherlock fMRI data used in this study included a scan in which participants viewed the first half (25 minutes) of the episode (Chen et al., 2016; Zadbood et al., 2017). In addition, a scanning session in which participants viewed the second half of the episode (Chen et al., 2016) was used for region of interest localization. CamCAN data used in this study included a scanning session of the short movie (8 min) Bang! You're Dead, and a resting-state scan that was used for region of interest localization.
Behavioral performance measures	Movie viewing and resting state scans in the fMRI were passive.

Acquisition

Imaging type(s)	functional
Field strength	3T
Sequence & imaging parameters	Sherlock fMRI data received from Chen et al. (2016) and Zadbood et al. (2017) consisted of preprocessed 3-T fMRI T2*-weighted echo-planar imaging (EPI) blood-oxygen-level-dependent (BOLD) responses with whole-brain coverage (TR 1,500 ms), in Montreal Neurological Institute (MNI) standard volume space. CamCAN data (Shafto et al., 2014; Taylor et al., 2017) used in this study consisted of 3-T fMRI T2*-weighted EPI raw BOLD responses with whole-brain coverage. Movie-scan TR was 2,470 ms, resting-state TR was 1,970 ms.
Area of acquisition	whole brain.

Diffusion MRI ☐ Used ☒ Not used

Preprocessing

Preprocessing software	Preprocessing was performed with MATLAB versions 2016, 2018, 2019 (MathWorks) with statistical parametric mapping (SPM12), on raw signals only (CamCAN data) and included slice-timing correction, spatial realignment, transformation to MNI space (voxel size 3 mm x 3 mm x 3 mm), and spatial smoothing with a 6 mm full-width at half-maximum (FWHM) Gaussian kernel. Thereafter, all data underwent voxel-wise detrending and z-scoring (demeaned and divided by standard deviation) across scan volumes.
Normalization	Linear transformation via SPM12.
Normalization template	All data were transformed to MNI space.
Noise and artifact removal	Motion correction (spatial realignment) via SPM12.

Volume censoring | n/a

Statistical modeling & inference

Model type and settings

We used inter-subject functional correlation (ISFC) analysis. For each participant we extracted the average across voxels within each region-of-interest (ROI), along the response time-course of the target-movie scan. We then calculated the average across all other participants (excluding reference participant) across ROI voxels. ISFC between two ROIs was calculated in a sliding time-window of 15 scanning volumes, as the Pearson correlation between the signal time-course of each participant in the first ROI, and the average time-course of all other participants in the second ROI. Correlation values were Fisher-transformed and averaged across participants, resulting in a single mean correlation value per window per ROI pair. This was repeated for every volume (±7 TR in time-window), yielding a single time-course of ISFC values per ROI pair, describing the fluctuation in coactivation among each pair of tested regions throughout movie events.

For the main analysis, we developed a method of state-fluctuation pattern analysis (SFPA), consisting of complementary analyses, which examined the correlation across temporal patterns of coactivation and behavior, and the coactivation corresponding to peak cognitive states. As these analyses were performed across the means of independent groups, for behavior and for coactivation, the temporal patterns of one modality serve as independent predictors for the other. First, for the correlation SFPA, we first down-sampled the ISFC time-course to match the behavioral time-course, by selecting the ISFC scores centered on each of the behaviorally-tested events in the movie. Thus, each event was assigned a single ISFC score calculated, as described in the previous section, across the 15-TR time-window centered around the behavioral event onset (event TR ±7). Very early or late events, with less than 7 TRs available for ISFC scoring before and after event onset, were discarded, resulting in 49 events for Sherlock, and 36 events for Bang! You're Dead. For each behavioral measure, we then calculated the Pearson correlation between the temporal pattern of cognitive state and the temporal pattern of corresponding ISFC scores, separately for each pair of ROIs. This resulted in a matrix of correlation coefficients. Second, for the peak-state SFPA, we examined the event-triggered ISFC during peak cognitive states. To this end, we first identified the top 5 peaks along the temporal patterns of cognitive states, for each behavioral measure separately. ISFC values for each region pair were z-scored (demeaned and divided by their standard deviation) across the time-course of the movie. We then averaged the ISFC z-scores across all network ROIs, and across the 5 peak events, within an event window of 29 time-bins centered around the event onset (event TR ± 14). This resulted in a time-course of mean network ISFC, describing the overall network coactivation corresponding to each type of peak cognitive state.

Effect(s) tested

Correlation SFPA effects were tested via permutation test, performed by random shuffling of the time-series of ISFC and correlating again with the cognitive state, repeated 1000 times, thus resulting in the null distribution for significance testing. Significance was determined at $p < 0.05$ (2-tailed) by testing the original correlation value against the permutation distribution. Because permutation testing was repeated per ROI pair, p values were corrected for multiple comparisons using the false detection rate (FDR). Effects of peak-state SFPA were tested via permutation test, performed to compare between the ISFC of each cognitive state relative to every other state. This was done by measuring the maximum absolute difference between ISFC mean across 5 randomly-selected events, and ISFC mean across an additional 5 randomly-selected events, repeated 1000 times. Significance at $p < 0.05$ (1-tail) was tested against a single critical threshold of difference, determined by the 95th percentile of the distribution of the maximum differences. In addition, we tested inter-network differences in peak-state SFPA in a repeated-measures ANOVA of ISFC with network (DMN, DAN, Vis) and cognitive state (surprise, emotional intensity, vividness, importance, episodic memory, emotional valence, theory of mind) as within-subject factors, as well as in a repeated-measures ANOVA of ISFC during peak surprise, with network as the only factor.

Specify type of analysis: ☐ Whole brain ☒ ROI-based ☐ Both

Anatomical location(s)

Functional network and ROI localization was performed on preprocessed data in two steps, constraining selection first by response correlation within tested participant sample, and second by previous functional network definitions based on vast samples. To measure response correlations within our current sample, we calculated the seed-based functional connectivity during rest and non-target movie scans, independent of the target movie data later used for inter-subject functional correlation (ISFC) analysis. Spherical 80-voxel seeds were defined anatomically in MNI space, for each of the 3 tested networks separately, based on locations validated in previous reports. Functional connectivity was calculated separately for each participant by correlating the signal time-course within each voxel with the average signal time-course of the seed region. Pearson coefficients within each voxel were averaged across participants, resulting in 3 correlation maps corresponding to the 3 seeds. Voxels with mean correlation values above predefined cut-off were included in the second selection step. The second selection step utilized a predefined parcellation of 17 functional networks, by discarding voxels outside the predefined networks from each of the corresponding correlation maps. Finally, remaining voxels were allocated to gross anatomical regions based on the atlas definition of each network.

Statistic type for inference (See Eklund et al. 2016) | non-parametric and ANOVA (see above)

Correction | False detection rate (FDR)

Models & analysis

n/a	Involved in the study
☐	☒ Functional and/or effective connectivity
☒	☐ Graph analysis
☐	☒ Multivariate modeling or predictive analysis

Functional and/or effective connectivity | Pearson correlations between regions and between subjects, using inter-subject functional correlation (ISFC;

Simony et al., 2016).

Multivariate modeling and predictive analysis	The multivariate patterns examined were the distribution of fMRI and behavioral responses across time. To clarify, we did not use any method of classification but rather a correlation across temporal patterns. See detailed explanation on our SFPA technique above.
